# Clinical implications of drug-screening assay for recurrent metastatic hormone receptor-positive, human epidermal receptor 2-negative breast cancer using conditionally reprogrammed cells

**DOI:** 10.1038/s41598-019-49775-w

**Published:** 2019-09-16

**Authors:** Rei Mimoto, Satomi Yogosawa, Hiroki Saijo, Atsushi Fushimi, Hiroko Nogi, Tadashi Asakura, Kiyotsugu Yoshida, Hiroshi Takeyama

**Affiliations:** 1 Department of Breast and Endocrine Surgery, The Jikei University School of Medicine, Tokyo, Japan; 2 Department of Biochemistry, The Jikei University School of Medicine, Tokyo, Japan; 3 Department of Anatomy, Jikei University School of Medicine, Tokyo, Japan; 4 Radioisotope Research Facilities, Jikei University School of Medicine, Tokyo, Japan

**Keywords:** Targeted therapies, Metastasis, Breast cancer

## Abstract

Various new drugs have been developed for treating recurrent hormone receptor-positive (HR+)/human epidermal receptor 2-negative (HER2−) breast cancer. However, directly identifying effective drugs remains difficult. In this study, we elucidated the clinical relevance of cultured cells derived from patients with recurrent HR+/HER2− metastatic breast cancer. The recently established conditionally reprogrammed (CR) cell system enables us to examine heterogeneity, drug sensitivity and cell function using patient-derived tumour samples. The results of microarray analysis, DNA target sequencing and xenograft experiments indicated that the mutation status and pathological features were preserved in CR cells, whereas RNA expression was different from that in the primary tumour cells, especially with respect to cell adhesion-associated pathways. The results of drug sensitivity assays involving the use of primary breast cancer CR cells were consistent with gene expression profiling test data. We performed drug-screening assays using liver metastases, which were sensitive to 66 drugs. Importantly, the result reflected the actual clinical course of this patient. These results supported the use of CR cells obtained from the metastatic lesions of patients with HR+/HER2− breast cancer for predicting the clinical drug efficacy.

## Introduction

Various new drugs, including a selective oestrogen receptor degrader (SERD), the mammalian target of rapamycin (mTOR) inhibitor, PI3K inhibitor and cyclin-dependent kinase (CDK)4/6 inhibitor, have been developed for recurrent hormone receptor-positive (HR+)/human epidermal receptor 2-negative (HER2−) breast cancer^1–5^. Conventional chemotherapy can also be used to treat patients with metastatic breast cancer. Although several studies investigated the association between responses to drugs and biomarkers using pathological, DNA mutation, or RNA expression analysis, it remains difficult to directly identify effective molecular targeted drugs and determine the best treatment sequence or combination for individual patients. If we test and identify effective drugs for each recurrent breast cancer type, we could select effective drugs for each patient and prevent unnecessary side effects.

To overcome this problem, several drug screening systems involving the use of patient-derived tumours have been established. Patient-derived xenografts closely recapitulate the genotype and phenotype of patient tumours^6^. They have also displayed a high degree of translatability to patients and provided an effective means for studying resistance mechanisms. However, the success rates are extremely low, including rates of 4–7% in HR+ breast cancer^7^. Furthermore, in this method, several months are required to perform drug testing. Next, cell culture systems established from metastatic lesions, such as tumour organoids and cancer tissue-originated spheroids (CTOSs), were investigated. Tumour organoids is a promising method and allows the long-term expansion of three-dimensional breast cancer cells from primary and metastatic sites with high success rates (>80%)^8–10^. However, the procedure has a learning curve and requires 2–6 months for establishment of the drug screening model. CTOSs are cultured in serum-free medium, which was developed to culture human embryonic stem cells^11,12^. However, the method requires the use of xenograft mice for passage and has not been effective for breast cancer to date.

Recently, Schlegel *et al*. demonstrated that conditional reprogramming cell system is a new cell culture technique for rapidly growing normal and tumour cells^13,14^. Conditionally reprogrammed (CR) cells are developed by co-culturing irradiated mouse fibroblast feeder cells with fresh epithelial cells in the presence of the Rho kinase inhibitor Y-27632. Concerning breast epithelial cells, CR cells could be generated from both normal epithelial and cancer cells^15–17^. The genomic and histological features of the original tumour are maintained in CR cells^17–19^. This method makes it possible to directly assess drug sensitivity and conduct molecular analysis in individual cancer patients. It has been reported that CR cells were useful for identifying effective treatments for respiratory papillomatosis^20^, adenoid cystic carcinoma^21^, pancreatic cancer^22^ and prostate cancer^23,24^. Inspired by these results, in this study, we aimed to reveal the characteristics of breast cancer CR cells and clarify whether drug sensitivity assays using CR cells taken from patients with HR+/HER2− metastatic breast cancer are useful for assessing drug efficacy in consideration of their potential clinical application.

## Results

### Generation of CR cells from small amounts of primary breast cancer tissue

**Figure 1:**
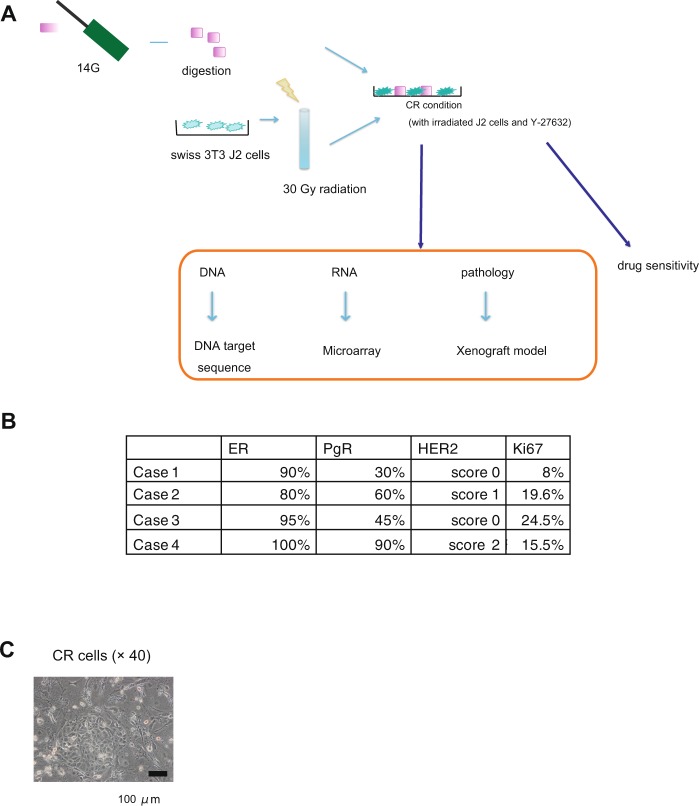
Generation of conditionally reprogrammed (CR) cells from small amounts of primary breast cancer tissue. (**A**) Workflow summarising the characterisation of CR cells of primary breast cancer via microarray analysis, DNA sequencing, histological analysis of the xenograft model and drug sensitivity assays. (**B**) The immunohistochemical characteristics of four patients with primary breast cancer. (**C**) Representative image of breast cancer CR cells. Scale bar indicates 100 μm.

We first attempted to generate CR cells from small amounts of primary breast cancer tissue (Fig. 1A). To avoid the effect of tissue collection on the pathological diagnosis of primary breast cancer, we performed ultrasound-guided core needle biopsy immediately after surgery or before neoadjuvant endocrine therapy. The collected tissue resected using a 14G needle was sufficient to generate CR cells. In four of five patients with HR+/HER2− tumours, CR cells could be produced within 2 weeks (Fig. 1B,C).

### Differences between the primary tumour and corresponding CR cells

**Figure 2:**
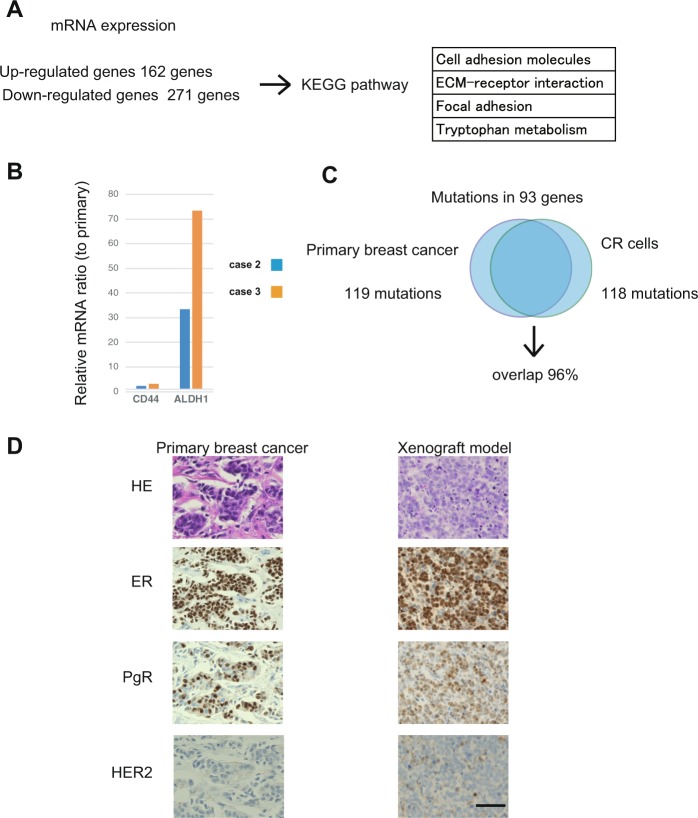
Differences between the primary tumour and corresponding conditionally reprogrammed (CR) cells. (**A**) Microarray analysis of primary breast tumours and CR cells. A total of 162 genes were identified to be up-regulated in CR cells compared with that in primary tumour, whereas 271 genes were detected to be down-regulated. KEGG pathway analysis was performed. (**B**) Relative mRNA expression ratio in CR cells compared with primary breast cancer tissues in case 2 and 3. (**C**) Differences in mutation status between primary breast tumours and CR cells for the 93 genes most commonly mutated in breast cancer. (**D**) Pathological analysis of the xenograft model. Upper panels, hematoxylin and eosin (HE) stained images. The expression of oestrogen receptor (ER), progesterone receptor (PgR) and human epidermal receptor 2 (HER2) was detected by via immunohistochemistry. The primary tumour images (left) corresponded to the xenograft tumour of CR cells (right). Scale bar indicates 50 μm.

We next performed microarray analysis of CR cells and primary breast tumours. The 162 genes were identified to be up-regulated in CR cells compared with that in primary tumour cells, whereas 271 genes were detected to be down-regulated. KEGG pathway analysis was performed with up-regulated and down-regulated genes. RNA expression analysis illustrated that RNA expression differed between CR cells and primary tumour cells, especially in cell adhesion-associated pathways (Fig. 2A). Stem-cell markers, such as CD44 and ALDH1, were up-regulated in CR cells of both case 2 and 3 (Fig. 2B). These data suggested that CR cells are reprogrammed and have undifferentiated features compared with the primary tumour cells. We next examined the DNA sequences of the 93 most commonly mutated genes in human breast cancer samples (Supplementary Table S2). In total, 119 mutations were detected in primary breast tumours, whereas 118 mutations were detected in CR cells. The overlap rate including mutation sites between CR cells and primary tumours was 96% (Fig. 2C). Moreover, the xenograft model had similar pathological features as the primary tumours (Fig. 2D). Oestrogen receptor (ER), progesterone receptor (PgR) and HER2 expression was preserved in CR cells in the *in vivo* experiment (Supplementary Table S1). These data suggested that the CR cell system is a good preclinical model for breast cancer.

### Drug sensitivity assay using CR cells

**Figure 3:**
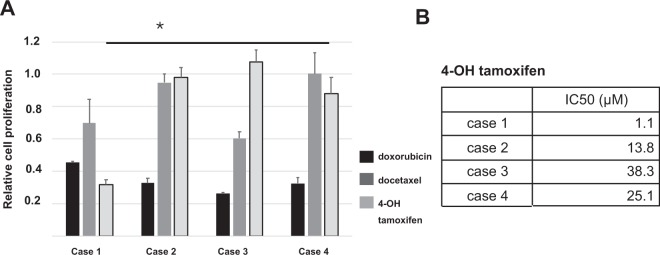
Drug sensitivity assay using conditionally reprogrammed (CR) cells from tumour. (**A**) Drug sensitivity assay of CR cell tumorspheres using doxorubicin, docetaxel and 4-OH-tamoxifen. Relative ratios compared with DMSO treatment are shown. The data are presented as the mean ± SD (n = 3; Student’s t-test, *P < 0.05). (**B**) IC50 of 4-OH-tamoxifen in four patients.

Next, we examined whether CR cells can be used in drug sensitivity assays. We generated CR cell tumorspheres in ultra-low attachment 96-well plates. Compared to cases 2 and 3, case 1 was more sensitive to endocrine therapy (Fig. 3A). The IC50 for 4-OH-tamoxifen was more than 10-fold lower in case 1 than in cases 2 and 3 (Fig. 3B). Clinically, the Onco*type* DX Recurrence Score was 17 in case 1, indicating a low risk of recurrence and permitting treatment using endocrine therapy alone. This result resembles the result of the *in vitro* drug sensitivity assay in case 1. Only case 4 received neoadjuvant endocrine therapy. We compared the clinical response to the drug sensitivity assay of CR cells. After administering endocrine therapy for 4 months, case 4 had SD. Consistent with clinical response, CR cells acquired before endocrine therapy were resistant to 4-OH tamoxifen (Fig. 3A,B). These data suggested that CR cells are useful for examining the clinical effectiveness of drug therapy.

### Drug screening using CR cells from recurrent metastatic breast cancer

**Figure 4:**
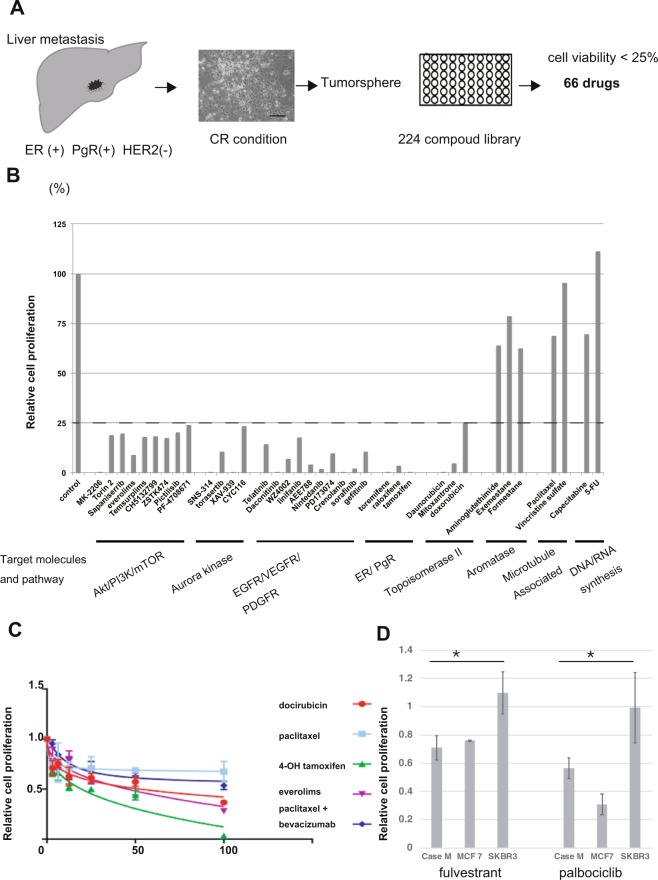
Drug-screening using conditionally reprogrammed (CR) cells generated from a patient with recurrent metastatic breast cancer. (**A**) A hormone receptor-positive (HR+)/human epidermal receptor 2-negative (HER2−) metastatic sample was obtained, and CR cells were generated (Case M). Diagram illustrating the steps involved in the drug-screening assay. The drug concentration was 10 μM. Using a cut-off value of 25%, 66 drugs were identified as effective for this patient. (**B**) Percent viability of CR cells following exposure to various compounds compared with the viability following DMSO treatment. Representative molecules and pathways targeted by the screened compounds are shown. (**C**) Drug sensitivity assay of CR cell tumorspheres using everolimus, doxorubicin, paclitaxel, paclitaxel plus bevacizumab and 4-OH-tamoxifen. Relative ratios compared with DMSO treatment are shown. Horizontal line shows the concentration of drugs (µM). (**D**) Drug sensitivity assay of CR cell tumorspheres using 1 µM palbociclib and 10 μM fulvestrant. Relative ratios compared with DMSO treatment are shown. The data are presented as mean ± SD (n = 3; Student’s *t*-test, *P < 0.05).

To evaluate the utility of CR cells for *in vitro* drug screening, we first generated CR cells from a site of recurrent metastasis. We acquired tissue from an ER+/PgR+/HER2− liver metastasis and cultured the tissue under CR conditions (Fig. 4A and Supplementary Table S4). More time was required to generate CR cells from the metastatic lesion compared with the observations for primary breast cancer. On day 7, two colonies were detected in a T25 flask. After 8 weeks, the number of CR cells was sufficient for drug screening. We performed drug screening using a cancer-related compound library. In total, 66 of 224 compounds reduced cell viability to less than 25% of that for the DMSO vehicle control (Supplementary Table S3). These effective drugs targeted several important pathways and molecules, including (1) PI3K/AKT/mTOR, (2) aurora kinase, (3) receptor tyrosine kinase, (EGFR, VEGFR, FGFR, and PDGFR), (4) ER/PgR and (5) topoisomerase II signalling. Conversely, some commonly used breast cancer drugs, such as aromatase inhibitors, microtubule-binding agents and DNA/RNA synthesis inhibitors, were ineffective (Fig. 4B). To confirm this phenomenon, we performed drug sensitivity assays using 4-OH-tamoxifen as a selective estrogen receptor modulator, doxorubicin as a topoisomerase II inhibitor, everolimus as mTOR inhibitor and paclitaxel as microtubule-binding agents. Tamoxifen, everolimus and doxorubicin were effective, whereas tumorspheres were resistant to paclitaxel (Fig. 4C). The sensitivity to paclitaxel was not increased by the addition of bevacizumab. Clinically, this patient received paclitaxel plus bevacizumab for 8 months before metastasectomy, and there were residual tumours in the liver. After metastasectomy, SERD and CDK4/6 inhibitors were used in this patient. No recurrence was noted at 13 months after metastasectomy. Consistent with clinical course, CR cells were more sensitive to these drugs than SKBR3 cells (HR-) and exhibited sensitivity similar that of MCF 7 cells (HR+) (Fig. 4D).

These results suggest that CR cells obtained from metastatic lesions of HR+/HER2− breast cancer respond to the drugs in accordance with the clinical outcome.

## Discussion

This is the first study to demonstrate the clinical relevance of CR cells for drug sensitivity test and drug screening of HR+/HER2− breast cancer.

With respect to clinical practice, the similarity between the results of drug sensitivity test and actual response to treatment was observed. First, the CR cells from neoadjuvant endocrine therapy resistant breast cancer were also found to be resistant to 4-OH-tamoxifen in drug testing. Second, the results of drug sensitivity assay suggested the patient was less likely to benefit from adjuvant chemotherapy. The gene expression assay Onco*type* DX test revealed low recurrence score, which means that the tumour may be effectively treated with endocrine therapy alone, thereby preventing the adverse effects of chemotherapy^25,26^. Corresponding CR cells were sensitive to endocrine therapy rather than chemotherapy. Third, Consistent with clinical course, CR cells from metastatic site showed resistance to paclitaxel plus bevacizumab and sensitivity to fulvestrant plus palbociclib. All these data supported the potential use of drug sensitivity testing for selecting therapy in both the perioperative chemotherapy and recurrent metastasis settings. However, we should consider the *in vivo* mechanism of each drug. For example, the effect of aromatase inhibitor could be evaluated only in *in vivo* setting. Because bevacizumab, anti-VEGF monoclonal antibody, inhibits the process of angiogenesis *in vivo*, it is difficult to examine its effect using *in vitro* drug-screening assay.

From the aspect of technical adjustment, we could easily and expeditiously obtain CR cells. We could perform (1) rapid drug screening with 8 weeks after biopsy (2) using a small sample amount obtained via 14G core needle biopsy. We also demonstrated (3) the effectiveness of X-ray irradiation of J2 cells. Considering the potential clinical application, the time until CR cells become available for sensitivity assays is important. Despite their slow growth, CR cells from patients with HR+/HER2− breast cancer could be obtained more rapidly than those from other patient-derived models. The amount of tissue required for establishing the model is also essential. We should avoid taking a large area of the tumour, as this can affect diagnosis. Biopsy using a 14G needle is relatively harmless and tolerable. In this study, X-ray is effective in place of caesium irradiation.

Previous research found that a similar number of copy number aberrations was observed between CR cells and primary tumours, with >95% overlap for the most frequently affected cytobands. The findings also revealed the retention of specific somatic variants in the CR cells as found in their original primary breast and prostate cancer tumours^17,23^. It has been demonstrated that CR cells maintain most of the intra-tumour heterogeneity of non-small cell lung cancer^18^. Characterisation of cells using DNA fingerprint analysis revealed identical patterns between tumour CR cells and xenograft models^27^. Consistently, our results indicated that CR cells largely maintained the mutation status of important genes compared with the tumours. Taken together, these results suggest the potential of CR cells approach for analysing the effect of specific mutation on drug resistance. Currently, CR cells are being applied in clinical trials in non-small cell lung cancer (EATON study; the information was obtained from Clinicaltrials.gov). Alternatively, the gene expression differences between CR cells and primary tumour cells were observed, particularly in cell adhesion-associated pathway. CD44 and ALDH1 expression were also increased in CR cells. Previous study also demonstrated that CR cells exhibit increased expression of several proteins that are up-regulated in adult epithelial stem cells, such as integrin α6, integrin β1, ΔNp63α, CD44, and hTERT^28^. Reprogramming is the primary process in CR cells. The epigenetic changes, such as DNA and histone modifications, alter gene expression patterns and regulate cell identity in the reprogramming of somatic cells^29^. Epigenetics changes caused by CR may result in rapid and dramatic differences in gene expression between primary tumour and CR cells.

A major limitation of this study was obviously the lack of cases. Collecting and analysing more samples are required for confirmation.

In summary, we generated CR cells from both primary and metastatic HR+/HER2− breast cancer tissue. We revealed the potential use of CR cells for drug sensitivity testing and treatment selection. Therefore, our study represents a key step in the development of novel clinical decision-making tools for metastatic HR+/HER2− breast cancer. To address emerging molecular targeted drugs, the clinical relevance of the sensitivity assay should be assessed in prospective clinical trials after the accumulation of further experiments.

## Methods

The Jikei University School of Medicine’s Ethics Review Committee approved the study protocol. All animal studies were approved by the Animal Care and Use Committee of the Jikei University School of Medicine. All methods presented here were performed in accordance with the relevant guidelines and regulations approved by Jikei University School of Medicine.

### CR cell culture

CR cells were generated from primary and metastatic breast cancer lesions. Immediately after surgical resection, we performed 14G core needle biopsy to obtain a minimal amount of tissue. One case had core needle biopsy before neoadjuvant endocrine therapy.

Acquired specimens were stored in ice-cold HBSS (Nacalai Tesque, Kyoto, Japan) until use. The biopsy specimens were cut into small pieces, collected via centrifugation and then digested in collagenase/hyaluronidase solution (Stemcell Technologies, Vancouver, BC, Canada) and dispase (Corning, NY, USA) at 37 °C for 2.5 h on a shaker. After dissociation, cell pellets were suspended in 10 ml of complete DMEM and filtered through a 100-μm cell strainer. Cells were then placed in T25 flasks layered with irradiated Swiss-3T3-J2 fibroblast feeder cells (Kerafast, Boston, MA, US) in the presence of a 10 μM Rho kinase inhibitor (Y-27632) (Wako, Osaka, Japan) in complete F-medium, as previously described^14^. Before co-culture, detached and collected J2 cells were resuspended in 10 ml of DMEM and irradiated with X-ray (30 Gy).

A total of 500 ml complete F-medium comprises 373 ml of complete DMEM (Nacalai Tesque),125 ml of F12 nutrient mix (Gibco, New York, USA), 0.5 ml of hydrocortisone/ EGF mix (Sigma-Aldrich, St Louis, MO, USA) (Invitrogen, Waltham, MA, USA), 0.5 ml of insulin (Sigma-Aldrich), 0.5 ml of amphotericin B (Lonza, Verviers, Belgium), 0.5 ml of gentamicin (Nacalai Tesque) and 4.3 μl of cholera toxin (Sigma-Aldrich). The solution was filter-sterilised using a 0.2-μm sterile filter. After adding the ROCK inhibitor Y-27632 at a final concentration of 10 μM, complete DMEM consists of 500 ml of DMEM, 50 ml of FBS (Cosmo Bio, Tokyo, Japan), 5.5 ml of l-glutamine (Nacalai Tesque) and 5.5 ml of penicillin-/streptomycin (Nacalai Tesque). Swiss-3T3-J2 cells were maintained in complete DMEM.

### Clinical analysis

Four breast cancer samples from surgically treated patients were obtained at Jikei University Hospital. The Jikei University School of Medicine Ethics Review Committee approved the study protocol, and informed consent was obtained from all patients. The pathological subtype was determined according to ER, PgR and HER2 expression. Onco*type* DX (Genomic Health, Redwood City, CA, USA) was performed using FFPE samples to estimate the benefit of chemotherapy. With RECIST 1.1, we utilised the following classifications for therapeutic response: complete response, primary tumour disappearance; partial response (PR), ≥30% decrease in the longest diameter of primary tumour; progressive disease (PD), ≥20% increase in longest diameter of primary tumour; and stable disease (SD), tumours that did not show either sufficient shrinkage to be classified as PR or sufficient increase to be classified as PD.

### Xenograft studies

All animal studies were approved by the Animal Care and Use Committee for Jikei University School of Medicine and conducted in accordance with the guidelines for animal experimentation established at Jikei University School of Medicine (Tokyo, Japan). For the xenograft model, 5 × 10^6^ CR cells in 100 µl of Matrigel (BD Bioscience, San Jose, CA, USA) were injected underneath the nipple of the mammary fat pad in the abdomen of 7-week-old female nude mice (Sankyo Lab, Tokyo, Japan). Simultaneously, these mice were implanted with pellets containing 17 beta-oestradiol (Innovative Research of America, Sarasota, FL, USA).

### DNA target sequence

DNA was extracted from primary breast tumours and CR cells using NucleoSpin® Tissue XS (Clontech, Mountain View, CA, USA). QIAseq Targeted DNA Panels (QIAGEN, Hilden, Germany) were used to analyse the genetic variants of the 93 most commonly mutated genes in human breast cancer samples (Supplementary Table S2).

### Microarray analysis

Total RNA from primary breast tumours and CR cells was purified using NucleoSpin® RNA XS (Clontech) and used for microarray analysis using a SurePrint G3 Human GE microarray kit Ver. 2.0, Agilent Technologies, Santa Clara, CA, USA). The results are available in the Gene Expression Omnibus database under accession no. GSE131081.

### KEGG pathway analysis

We used the results of no. GSE131081 for KEGG enrichment analysis, which was performed using The Database for Annotation, Visualisation and Integrated Discovery Ver. 6.7 (http://david.abcc.ncifcrf.gov/summary.jsp).

### Drug sensitivity assay

One hundred CR cells in 100 µl of complete F-medium were seeded in ultra-low attachment 96-well plates (Corning) to generate tumorspheres. The tumorspheres were treated with DMSO, 4-OH-tamoxifen (Sigma-Aldrich), doxorubicin (WAKO), docetaxel (WAKO), paclitaxel (WAKO), bevacizumab (Selleck), fulvestrant (Selleck) or palbociclib (Selleck) and incubated at 37 °C in 5% CO_2_. After 48 h of treatment, the CellTiter-Glo® 3D cell viability assay (Promega, WI, USA) was used to assess growth inhibition. To determine the IC50, cells were treated for 4 days with 4-OH-tamoxifen. was also used for drug sensitivity assay.

### Drug-screening assay

The Cambridge Cancer Compound library was purchased from Selleck Chemicals (Houston, TX, USA). After generating tumorspheres in 96-well plates, screening compounds were added to the plates at a final concentration of 10 μM. Sensitivity was assessed after 36 h of treatment using the CellTiter-Glo® 3D cell viability assay.

### Statistical analysis

Statistical analyses of the continuous variables were performed using a two-tailed Student’s *t*-test. Data are presented as the mean ± SD. *P* < 0.05 denoted statistical significance. All statistical analyses were performed using Stata Statistical Software (release 13.0; Stata Corp).

## Supplementary information

Supplementary information

## Data Availability

The data that support the findings of this study are available from the corresponding author on request.
