# Supplementary material for: Clinical implications of drug-screening assay for recurrent metastatic hormone receptor-positive, human epidermal receptor 2-negative breast cancer using conditionally reprogrammed cells
Source: Sci Rep. 2019 Sep 16;9:13405. doi: 10.1038/s41598-019-49775-w (PMC6746954; doi:10.1038/s41598-019-49775-w)
Supplement: Supplementary file 1 — Supplementary information [file 41598_2019_49775_MOESM1_ESM.docx]

**Clinical implications of drug-screening assay for recurrent metastatic hormone receptor-positive, human epidermal receptor 2-negative breast cancer using conditionally reprogrammed cells**

Rei Mimoto^1＊^, Satomi Yogosawa^2^, Hiroki Saijo^3^, Atsushi Fushimi^1^, Hiroko Nogi^1^, Tadashi Asakura^4^, Kiyotsugu Yoshida^2^, Hiroshi Takeyama^1^

1 Department of Breast and Endocrine Surgery, Jikei University School of Medicine

2 Department of Biochemistry, Jikei University School of Medicine

3 Department of Anatomy, Jikei University School of Medicine

4 Radioisotope Research Facilities, Jikei University School of Medicine

Supplementary table S1: Immunohistochemical analysis of the xenografted tumour.

|  | ER | PgR | HER2 |
| --- | --- | --- | --- |
| Case 1 xeno | 90% | 20% | score 0 |

Supplementary table S2: 93 genes which assessed the mutation by DNA sequence.

| ACVR1B | CDKN2A | GEN1 | NCOR1 | SEPT9 |
| --- | --- | --- | --- | --- |
| AKT1 | CHEK2 | HERC1 | SMAD4 | SMAD4 |
| APC | CSMD1 | HOXB13 | NF1 | SMARCA4 |
| AR | CTNNB1 | IRAK4 | PALB2 | STK11 |
| ATM | DIRAS3 | ITCH | PALLD | SYNE1 |
| ATR | EGFR | KMT2C | PBRM1 | TGFB1 |
| AXIN2 | EP300 | KRAS | PCGF2 | PCGF2 |
| BAP1 | EPCAM | MAP2K4 | PIK3CA | TRAF5 |
| BARD1 | ERBB2 | MAP3K1 | PIK3R1 | VHL |
| BLM | ERBB3 | MDM2 | PMS1 | WEE1 |
| BMPR1A | ERCC4 | MED12 | PMS2 | XRCC2 |
| BRCA1 | ESR1 | MEN1 | PPM1L | XRCC3 |
| BRCA2 | EXOC2 | MLH1 | PTEN | ZBED4 |
| BRIP1 | EXT2 | MRE11A | PTGFR |  |
| CASP8 | FAM175A | MSH2 | RAD50 |  |
| CBFB | FANCC | MSH6 | RAD51 |  |
| CCND1 | FBXO32 | MUC16 | RAD51C |  |
| CDH1 | FGFR1 | MUTYH | RAD51D |  |
| CDK4 | FGFR2 | MYC | RB1 |  |
| CDK6 | GATA3 | NBN | RET |  |

Supplementary table S3: 66 drugs suppressed cell viability of CR cells of metastatic breast cancer.

| Crisotinib | isponesob | AG-14361 |
| --- | --- | --- |
| YM155 | disulfiram | Fingolomod |
| XAV-939 | ponatinib | NVP-BSK805 |
| SNS-314 | tamoxifen | SFI-794833 |
| PHA-665752 | azd7762 | Volasertib |
| SRT1720 | toremifene | Daunorubicin |
| Alvespimycin | befetinib | Daconitinib |
| BI 2536 | doxercalcifero | Crenolanib |
| MK-2206 | raloxifene | Rabusertib |
| Nintedanib | celecoxib | gossypol acetic acid |
| Obatociax | AEE788 | Ixazomib |
| Sorafenib | aprepitant | Mitoxantrone |
| JNJ-268541 | tipifamib | TAME |
| Navitoclax | PD173074 | Rebastinib |
| WZ4002 | BX795 | Telatinib |
| Everolims | linifanib | CEP-33779 |
| Gefitinib | PF-4708671 | Silmitaserib |
| torasertib | doxerubicibe | CH5132799 |
| Nilotinib | Torin 2 | Linsitinib |
| BIIB021 | BIIB021 | Sapaniserrib |
| Temsurplims | Dasatinib | Pictilisib |
| CYC116 | Luminespib | Trichostatin A (TSA) |

|  | ER | PgR | HER2 | Ki67 |
| --- | --- | --- | --- | --- |
| Case M | 90% | 0% | score 0 | 7% |

Supplementary table S4: The immunohistochemical characteristics of three patients with primary breast cancer.
